# A distribution information sharing federated learning approach for medical image data

**DOI:** 10.1007/s40747-023-01035-1

**Published:** 2023-03-29

**Authors:** Leiyang Zhao, Jianjun Huang

**Affiliations:** Guangdong Key Laboratory of Intelligent Information Processing, College of Electronics and Information Engineering, Shenzhen University, Shenzhen, China

**Keywords:** Federated learning, Distribution information sharing, Image classification, Non-IID

## Abstract

In recent years, federated learning has been believed to play a considerable role in cross-silo scenarios (e.g., medical institutions) due to its privacy-preserving properties. However, the non-IID problem in federated learning between medical institutions is common, which degrades the performance of traditional federated learning algorithms. To overcome the performance degradation problem, a novelty distribution information sharing federated learning approach (FedDIS) to medical image classification is proposed that reduce non-IIDness across clients by generating data locally at each client with shared medical image data distribution from others while protecting patient privacy. First, a variational autoencoder (VAE) is federally trained, of which the encoder is uesd to map the local original medical images into a hidden space, and the distribution information of the mapped data in the hidden space is estimated and then shared among the clients. Second, the clients augment a new set of image data based on the received distribution information with the decoder of VAE. Finally, the clients use the local dataset along with the augmented dataset to train the final classification model in a federated learning manner. Experiments on the diagnosis task of Alzheimer’s disease MRI dataset and the MNIST data classification task show that the proposed method can significantly improve the performance of federated learning under non-IID cases.

## Introduction

In traditional medicine, imaging doctors need to perform many diagnostic classifications of images, which consumes considerable time and energy and may cause misjudgment due to environmental and other factors. In recent years, with the development of artificial intelligence related technologies, medical imaging disease diagnosis classification based on deep learning has gradually become one of the most promising areas for artificial intelligence due to its excellent performance.

In reality, deep learning requires large amounts of training data to achieve better performance. However, the quantity of imaging data available in a single hospital is small. Since the data contain private patient information, it is not possible to collect and organize data from multiple hospitals, which makes centralized machine learning impossible. Recently, federated learning (FL) has emerged as a new paradigm for distributed machine learning [1], which can jointly train a deep learning model with multiple data owners without data going out of local. Depending on the application scenarios, federated learning can be broadly categorized into cross-device FL and cross-silo FL, and the clients in the cross-silo setting are a small number of organizations (e.g., medical institutions) with reliable communications and abundant computing resources in datacenters [2]. We focus on cross-silo federated learning in this paper. Federated learning has been studied in medical applications such as health care [3, 4], automatic classification of medical images [5], MRI reconstruction [6], and COVID-19 detection [7, 8].

However, non-IID data present a serious challenge to federated learning. In the case of non-IID, the basic assumption of independent homogeneous distribution in federated learning will no longer be satisfied, and the optimization direction of the local model may be far from the global optimization direction, which inherently induces a local optimum [9]. Some algorithmic-level federated learning approaches to solve non-IID problems have recently emerged [9, 10]. However, Li et al. [11] shows that these methods perform as poorly as FedAvg [12] on image datasets with deep learning models. Recently, some data-level federated learning approaches emerge as new directions for solving non-IID problems [13, 14], but they can cause problems such as privacy leakage.

To better address the problem of degraded federated learning performance under non-IID image data, FedDIS is proposed. First, the client learns the distribution of all client datasets, augments each client dataset to reach IID according to the distribution, and finally trains the task model on the augmented dataset to solve the non-IID problem.

The main contributions of this study include the following:

1. We propose a distribution information sharing strategy that can preserve patient privacy to improve the performance of federated learning on non-IID data sets. In this method, each client shares its individual local data distribution in the hidden space for other clients to construct an IID dataset to solve the non-IID problem.
2. We use $\sigma$-point sampling to generate more hidden space data points to enable more reliable distribution parameter estimation and better privacy protection.
3. Metrics such as distance and maximum structural similarity between sets are introduced to measure the privacy-preserving ability of the proposed method.
4. We employ the difference in training performance between the generated data and the original to evaluate the effectiveness of the hidden space distribution information sharing strategy.
5. We conducted experiments to investigate the algorithm itself, including exploring the effects of different hidden space distribution types and $\sigma$-point parameters on the performance of the method.
6. We conducted comparative experiments to validate the superiority of the algorithm. The experiments consisted of two parts, which verified the advantages of the method under the cases of local data imbalance on the client side and inconsistent data distribution among the clients, respectively. The comparison results show that our method has better performance and faster convergence.

The remainder of this paper is organized as follows. “Related work” presents some related work. In “Methodology”, the overall process of the method is introduced, as well as the core distribution information sharing and data generation methods. “Experiments and results” gives the details of the experimental design and results and provides a comparative analysis of the experimental results. “Conclusion and future work” concludes the paper.

## Related work

In recent years, FL has emerged as a new paradigm for distributed machine learning; because of its secure and collaborative features, it has been widely used in medical and financial institutions. The problem of non-IID data causing degradation of federated learning performance is one of the main challenges for federated learning [15], and there are two types of solutions: data level and algorithm level.

At the data level, there are data-based methods and data-free methods. In the data-based approach, a globally shared small IID dataset can reduce the impact of non-IID data [16], but for fields such as medicine, data sharing is difficult to implement in practical applications. Sun et al. [17] introduced a data redundancy strategy to deal with non-IID data by exchanging local data with their trusted nodes to improve classification task accuracy in non-IID conditions. KD is a technique that teaches knowledge from one or more teacher models to an empty student model. FEDDFUSION [13] is a data-based KD approach that uses generated data to aggregate heterogeneous knowledge from all received client models. For the data-free approach, FEDGEN [14] is a data-free KD approach. The server learns a generator in a data-free manner and then broadcasts it to the client to adjust the training of the local model. At the algorithm level, [12] FedAvg was the first federated learning algorithm that emerged to deal with non-IID data to a certain degree. Wang et al. [18] used the relationship between the gradient magnitude and sample quantity to estimate the data class imbalance, and a new loss function, ratio loss, was designed to increase the effect of minority class data on the results. Sarkar et al. [19] improved the performance under federated learning class imbalance by reshaping the cross-entropy loss, reducing the weight of majority class data on the model when the model has high prediction accuracy for the majority class and increasing the weight of minority class on the model accordingly. FedProx [10] uses the global model to correct the local training direction; however, related studies have concluded that FedProx has little advantage over FedAvg.

Federated learning methods under non-IID data are becoming increasingly abundant; however, the cross-silo scenario is not well studied. Data-based approaches are limited in their application due to the risk of privacy breaches. The data-free approach is a new approach for solvingdime the non-IID data problem in federated learning due to its privacy-preserving ability and good performance, but the performance degradation problem on non-IID image data under federated learning has not been well studied on data-free methods.

## Methodology

### Problem description

A large quantity of medical data is needed to train a disease classification model; however, medical data are scattered in different medical institutions and cannot be collected centrally due to privacy issues. Let $X_1,...,X_m$ denote the local image dataset of m medical institutions. The corresponding data distribution is denoted as $P_1,...,P_m$; thus, $X=X_1\cup ...\cup X_m$ is the global dataset, and $P_X$ is the global data distribution. Assuming $X_i \cap X_j=\emptyset$ for $i\ne j$, let $|X_i|$ denote the cardinality of $X_i$. Assume a task model $f({\textbf {w}},{\textbf {x}},{\textbf {y}})$(e.g., a medical image classification model) is to be trained, and its corresponding model parameter is ${\textbf {w}}\in R^{d}$. Let $(x_j,y_j)$ denote a data sample; here, $x_j$ represents the input (image) to the task model, and $y_j$ represents its corresponding output (class label). Then, the global loss function $F({\textbf {w}})$ can be

1 $$\begin{aligned} F({\textbf {w}})=\frac{1}{|X|}\sum _{i=1}^m\left| X_i\right| F_i({\textbf {w}}), \end{aligned}$$

where $F_i({\textbf {w}})$ is the local loss function of client i, which is defined as

2 $$\begin{aligned} F_i({\textbf {w}})=\frac{1}{|X_i|}\sum _{j \in X_i}f({\textbf {w}},x_j,y_j). \end{aligned}$$

Training the task model under IID conditions can obtain good performance with FedAvg, and IID data in federated learning satisfy

3 $$\begin{aligned} P_i({\textbf {x}},{\textbf {y}})=P_X({\textbf {x}},{\textbf {y}}) \end{aligned}$$

where $P_i({\textbf {x}},{\textbf {y}})$ is the data distribution of the *i*-th client for *i* = 1...,*m* and $P_X({\textbf {x}},{\textbf {y}})$ is the global distribution. However, FedAvg suffers from performance degradation in the non-IID data case, i.e. $P_i({\textbf {x}},{\textbf {y}})\ne P_j({\textbf {x}},{\textbf {y}})$, a natural idea is to construct an augmented dataset $D_i$ for each client to make its distribution identical to others distributions, such that

4 $$\begin{aligned} X_i^{\prime }=X_i \cup D_i \Rightarrow P_i(\textbf{x}, \textbf{y})=P_j(\textbf{x}, \textbf{y}) \end{aligned}$$

where $D_i=\left\{ z \mid z \sim \sum _{j \ne i} P_j(\textbf{x}, \textbf{y})\right\}$ can be obtained by generative neural network learning such as VAE.

### Method

Data sharing and data redundancy strategies can improve the performance of federated learning under non-IID conditions [16, 17]. The data sharing strategy distributes a subset $X_G$ that obeys the global distribution $P_X$ to each client, and then the data of each client become $X_1\cup X_G,...,X_m\cup X_G$. The data redundancy strategy stores the data of each client in K different workers that will execute federated learning; for example, when there are two clients in total and K is 2, the data stored by the two workers is $X_1\cup X_2,X_2\cup X_1$. The sharing or exchange of raw data in the above methods is not applicable in most real scenarios because this may leak data privacy. Local data generation does not leak data privacy, such as the SMOTE method, augmenting the local dataset $X_i$ by obtaining a synthetic sample set $X_{S_i}$ by sampling at the nearest neighbors of a minority class of samples to obtain a new local dataset $X_i\cup X_{S_i}$ for model training, but this method is not effective due to its inability to utilize other clients’ data distribution; it does not change the degree of non-IID of each client data.

We improve the performance of federated learning under non-IID data through indirect data sharing and construct a dataset locally on the client with the same distribution as the global data. The process of FedDIS is divided into two stages: the first one is shown in the algorithm, and the second is to execute federated learning on each client using the FedAvg algorithm.
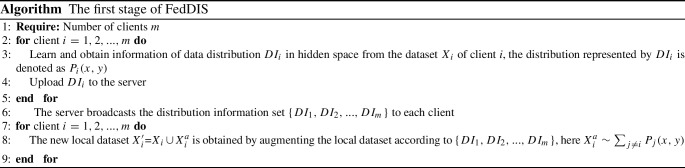


**Fig. 1:**
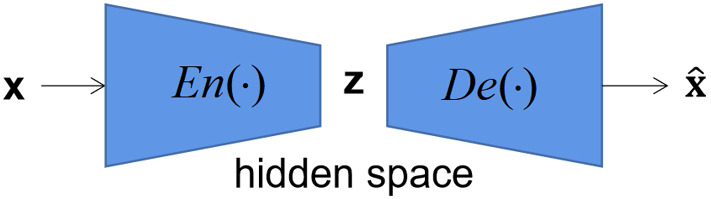
Simple structure of VAE

VAE has a good ability to learn data distributions [20]. To learn the distribution of high-dimensional medical data, we use the encoder in VAE to reduce the dimension of the original data and estimate the distribution of the encoded data in the hidden space, which does not disclose the privacy of the original data and reduces the communication cost of federated learning by reducing the quantity of data of distribution information. Let the VAE encoder and decoder be $En(\cdot )$ and $De(\cdot )$, respectively. The simple VAE structure is shown in Fig. 1.

In the implementation, the VAE is trained first, and then the $En(\cdot )$ is used to map the local data to the hidden space. To share distribution information (*DI*) of data, the local data are assumed to obey some probability distribution $p(\textbf{z}| {\theta })$ with parameters ${\theta }$ in the hidden space, ${\theta }$ is estimated from the hidden space data, *DI* contains the distribution type and ${\theta }$ of $p(\textbf{z}| {\theta })$, which is shared with other clients. Finally, the client augments the local data using its received distribution parameters and the decoder.

### Data augmentation based on sharing of hidden space distribution

Our core approach is to obtain information about the hidden space distribution of an individual client’s local dataset. A client encodes the local data into the hidden space, estimates the distribution parameters based on the reparameterized data and the distribution type, and shares them with other clients. Assume the distribution of the hidden space of a client is $p(\textbf{z}| {\theta })$; here, $\textbf{z}$ is the hidden space random vector of *d*-dimension, and ${\theta }$ is the parameter of the distribution. To estimate ${\theta }$, a set of reparameterized samples are generated by using the local images of the client and VAE encoder. Let the original image dataset of the client be $X=\{ \textbf{x}_i|i=1,2,...,k\}$; for each image $\textbf{x}_i$ in *X*, the VAE encoder gives

5 $$\begin{aligned} \left[ \begin{array}{c} \textbf{m}_i \\ \varvec{\Sigma }_i \end{array}\right] =E n\left( \textbf{x}_i\right) \end{aligned}$$

where $\textbf{m}_i$ is a *d*-dimensional vector and $\mathbf {\Sigma }_i$ is a $d\times d$ matrix. Since the resampling of VAE is such that $\textbf{u}\sim N(0,I)$ is first generated and then $\textbf{z}=\textbf{m}_i+\mathbf {\Sigma }_i\textbf{u}$ is made to be a sample of the hidden space, this is likely to produce wild values and only one sample of the hidden space is generated for each data sample, and the small number cannot be used well to estimate the distribution parameters. To obtain a more accurate estimation of the hidden space distribution parameters, reparameterization in the hidden space is conducted by using the $\sigma$-point sampling method [21] to generate a set of 2*d* $\sigma$-points as

6 $$\begin{aligned} \left\{ \begin{array}{l} \textbf{z}_{i, n}=\textbf{m}_i+\alpha \left( \sqrt{\varvec{\Sigma }_i}\right) _n, n=1,2, \cdots , d \\ \textbf{z}_{i, n}=\textbf{m}_i-\alpha \left( \sqrt{\varvec{\Sigma }_i}\right) _n, n=d+1, d+2, \cdots , 2 d \end{array}\right. \end{aligned}$$

where $\left( \sqrt{\varvec{\Sigma }_i}\right) _n$ is the *n*-th column of the square root of $\mathbf {\Sigma }_i$ and $\alpha >0$ is a constant that defines the exact placement of sigma points. The set of reparameterized samples in the hidden space will be

7 $$\begin{aligned} Z=\{ \textbf{z}_{i, n}|i=1,2,...,k;n=1,...,2d \} \end{aligned}$$

which is used to estimate ${\theta }$ of the selected distribution model $p(\textbf{z}| {\theta })$. Since the quantity of hidden space data generated using $\sigma$-point sampling is increased, the distribution of the hidden space can be better estimated, while the risk of privacy leakage of the original data samples is reduced because only at the encoded mean vector can the decoder recover data that are similar enough to the original data. When the distribution parameters are estimated, client *i* sends the distribution type, distribution parameters, and the number of local data elements $|X_i|$ to the server, which broadcasts them to other clients.

Once a client *k* receives the type of hidden space distribution and corresponding parameters of other clients from the central server, a set of hidden space samples $Z^{\prime }=\left\{ \textbf{z}_j^{\prime }|j=1,2, \cdots , \sum _{i \ne k}| X_i \mid \right\}$ can be generated to augment the training data

8 $$\begin{aligned} \textbf{x}_j^{\prime }=De(\textbf{z}_j^{\prime }) \end{aligned}$$

This augmented dataset

9 $$\begin{aligned} X^{\prime }=\left\{ \textbf{x}_j^{\prime }\left| j=1,2, \cdots , \sum _{i \ne k}\right| X_i \mid \right\} , \end{aligned}$$

which generates the corresponding amount of synthetic data according to the $|X_i|$ of each other client, $X^{\prime }$ is then used together with the original dataset to train the client’s local task model.

To explore the privacy preservation feature of the proposed method, the distance between the original dataset and the generated dataset is used, which is defined as follows:

10 $$\begin{aligned} d(A,B)=inf\{d(\textbf{x},B);\textbf{x}\in A \} \end{aligned}$$

where A is the generated dataset, B is the original dataset, and $d(\textbf{x},B)$ is the distance from point **x** to set B, which is defined as follows:

11 $$\begin{aligned} d(\textbf{x},B)=inf\{d(\textbf{x},\textbf{y});\textbf{y}\in B \} \end{aligned}$$

where $d(\textbf{x},\textbf{y})$ is the distance between **x** and **y** and is defined as follows:

12 $$\begin{aligned} d(\textbf{x},\textbf{y})=||\textbf{x}-\textbf{y}||_2 \end{aligned}$$

Table 1 shows the distance between the generated dataset and the original dataset under the uniform distribution type.

**Table 1:** Distance between the generated dataset and the original dataset

Dimension of dataset	Distance
1	3.0279e$-$05
2	0.0238
4	1.0769
8	4.8764

The table shows that as the dimensionality of the data increases, the distance increases, and the risk of privacy leakage decreases. Therefore, for high dimensional data, the possibility of privacy exposure of the data generated with VAE is small.

For privacy protection on real image datasets, structural similarity (SSIM) is used to show that generating data samples does not reveal individual private information in the original data samples. The maximum SSIM between the generated image set and the original image set is employed as a privacy-preserving metric, which is mathematically represented as

13 $$\begin{aligned} SSIM(A,B)=max\{SSIM(x,y);x \in A;y \in B\} \end{aligned}$$

**Fig. 2:**
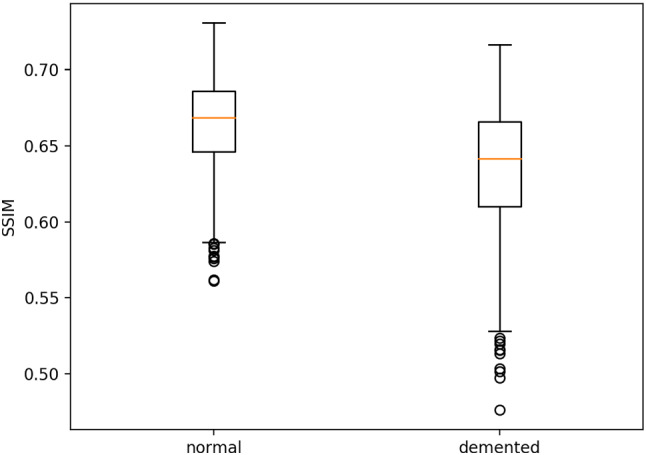
The boxplot of SSIM shows that the mean value is only approximately 0.65 and the maximum value is less than 0.75; thus, the privacy of the original data can be well protected

**Fig. 3:**
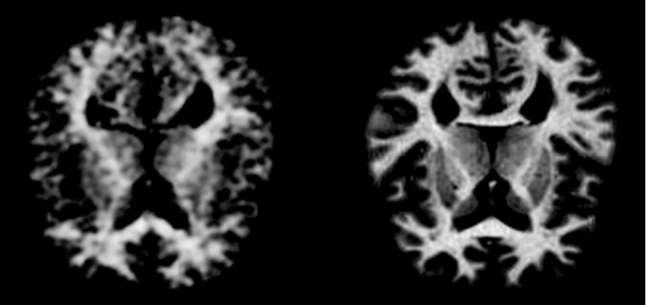
The chart with the largest SSIM of 0.73 shows that the texture difference between the generated image and the original image is very large, so the generated image does not leak the privacy of the original data

Fig. 2 shows the SSIM in an MRI dataset with 1000 generated samples. It can be seen that the SSIM of the generated data samples and the original data samples are very low, so the generated data are well secured by the original data. To more intuitively show the difference between the data generated by the distribution information and the original data, the diagram with the largest SSIM is shown in Fig. 3.

The method uses only the distribution of the dataset and does not involve individual data samples, so augmented datasets cannot disclose patient privacy, but they have the same distribution as the original dataset and, therefore, have similar effects on model training. We test with a classification task on an MRI dataset. The difference in training performance between the generated data and the original (Dpgo) after the final convergence of the individually trained models for the original and generated data is shown in Table 2. Dpgo is defined as follows:

14 $$\begin{aligned} {\text {Dpgo}}{} & {} \left( X, X^G\right) \nonumber \\{} & {} =\left\| \mathbb {E}_{x \sim X}[l(f(\textbf{w}, x, y))]-\mathbb {E}_{x \sim X^G}[l(f(\textbf{w}, x, y))]\right\| \nonumber \\ \end{aligned}$$

where *X* is the distribution of the original dataset, $X^G$ is the distribution of the generated dataset, *x* is the real training sample, *y* is the corresponding label, $\textbf{w}$ is the parameters of the task model, and $l(\cdot )$ is the cross-entropy loss function.

**Table 2:** Dpgo after training convergence

Avg	Min	Max
3.12e$-$06	0	1.65e$-$05

From the above results, we can see that the VAE-generated data have similar training effects as the original data.

## Experiments and results

In this section, we do two types of experiments. The first type of experiments is a research on the algorithm itself, including exploring the effects of different hidden space distribution types and $\sigma$-point parameters on the performance of the method, with the purpose of discovering which factors in the method can have an impact on the performance of the method. The second type of experiments is the comparison experiment, which consists of two parts, one is conducted under the condition of local data imbalance in the client, and the other is conducted under the condition of inconsistent data distribution among clients, and the purpose of the experiment is to verify the superiority of the method.

### Experimental setup

**Fig. 4:**
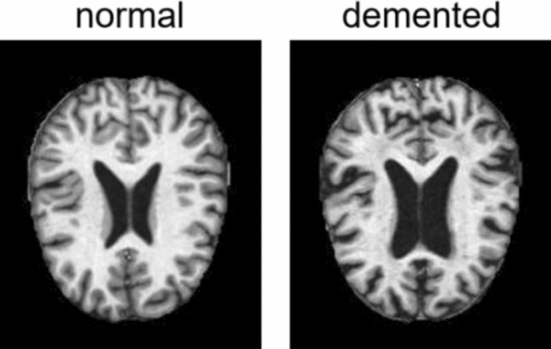
Sample image data for individuals with dementia and normal individuals

**Dataset: **The datasets used in the experiment include the Alzheimer’s disease MRI dataset and the MNIST dataset, the former is used for disease diagnosis, the later is used for digit image classifications. The original MRI dataset downloaded from Kaggle was divided into two parts, the training set and the test set, each containing four classes of data, namely, nondementia, very mild dementia, mild dementia, and moderate dementia, and data with dementia were integrated into one category. The integrated training set contains 2,561 data with dementia and 2,560 data without dementia, and the test set contains 639 data with dementia and 640 data without dementia. The original size of the images was 1$\times$176$\times$208, and they were resized to 1$\times$176$\times$176 during training and normalized. Sample image data for demented and normal individuals are shown in Fig. 4.

**Table 3:** Data partition

Data partition	Nondementia	Dementia
Case 1	2/10	6/10
	2/10	2/10
	6/10	2/10
	1/10	8/10
Case 2	1/10	1/10
	8/10	1/10

**Non-IID settings: **For MRI dataset, it was randomly partitioned according to Table 3 and assigned to different clients to give an example of federated learning under the condition of non-IID between hospitals in a real scenario, and EMD is used to measure the degree of non-IID. Let the data distribution of the three clients be denoted as p1, p2, and p3. Table 4 shows the EMD of the data between the three clients two by two. To better compare the results after expanding the non-IID dataset with those trained directly on the IID training set, we distribute the data equally to three clients as the data IID case. In the first division, the disease-free data accounted for 20%, 20% and 60% of the total disease-free training data, and the disease data accounted for 60%, 20% and 20% of the total diseased training data for the three clients. In the second division, the disease-free data of the three clients account for 10%, 10%, and 80% of the total disease-free training data, and the disease data account for 80%, 10%, and 10% of the total disease training data, respectively. Finally, the test set divided by the original dataset is used for performance evaluation, and the same test set is used on different clients to ensure fairness. For MNIST dataset, it was partitioned according to the Dirichlet distribution [22], the concentration parameter $\beta$ was set to 0.05, 0.1 and 1, respectively, in which a smaller $\beta$ indicates higher data heterogeneity, the number of clients was set to 20 with an active-user ratio r = 50%.

**Table 4:** EMD of the data between client pairs

Data partition	(p1, p2)	(p1, p3)	(p2, p3)
Case 1	0.606	0.641	0.601
Case 2	1.276	1.189	1.137

**Fig. 5:**
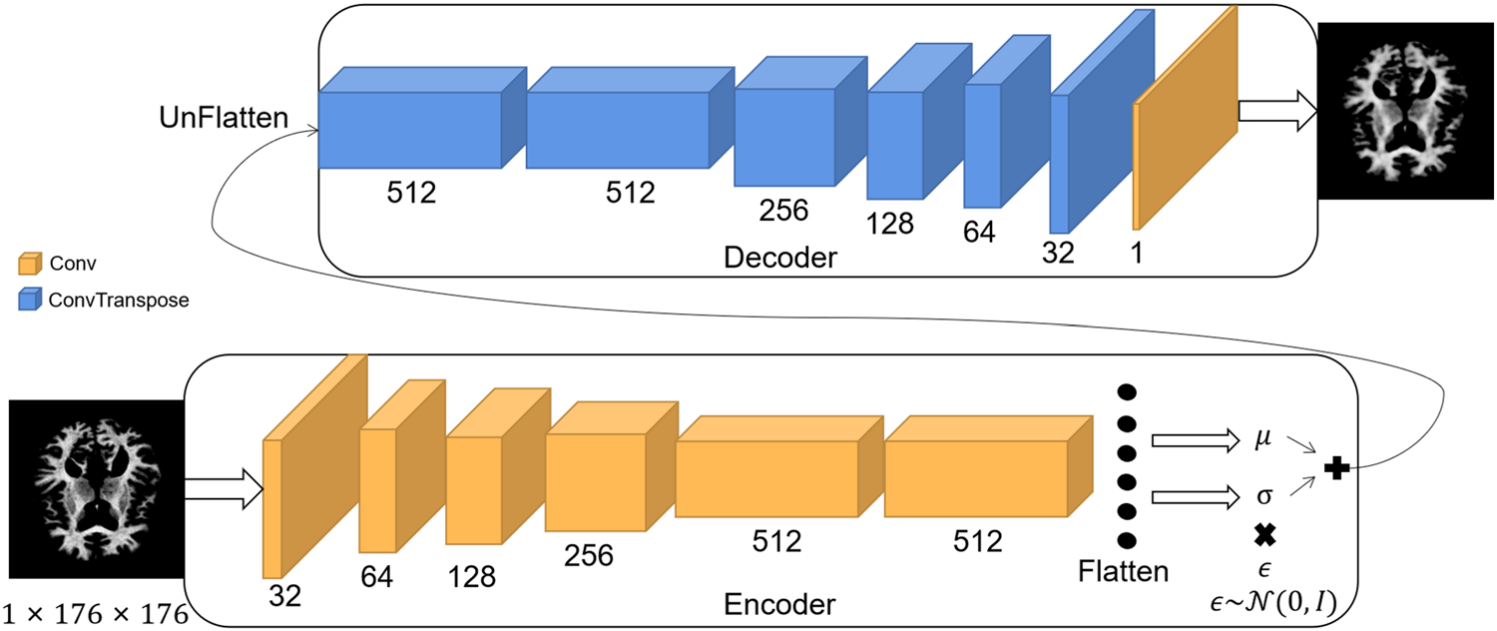
Structural diagram of the VAE model

**VAE model: **VAE is widely used in the processing of images [23–26], we designed a specific VAE model based on the Alzheimer’s disease dataset with the structure diagram shown in Fig. 5. The main structure of the model is divided into two parts, encoder and decoder, where the encoder maps the image data into mean and variance vectors and uses the reparameterization trick [20] to obtain the hidden variables. The reparameterization technique allows the model to perform the computation of gradients and backpropagate the error from the decoder to the encoder, thus making the whole model trainable. To speed up the training and reduce the communication cost of federated learning, BatchNorm is used between all convolution and transposed convolution layers, and we use LeakyReLU as the activation function. For MNIST, we adjusted the parameters of the VAE model to fit the format of the MNIST data.

**Task model: **The popular neural network VGG16 was used to perform diagnostic tasks on MRI dataset. To train the network faster and minimize the communication cost of federated learning, we use a transfer learning technique that utilizes the pretrained model that comes with the PyTorch framework. It is a classification network with 1000 categories trained on ImageNet with more than 1.2 million images. Although these images are from the natural scene domain, the images generally share some low-level features, such as lines, textures, and edges. Since our dataset is a single-channel image and the classification task is 2 classifications, we replace the input of the first convolutional layer with a single channel and the output of the last fully connected layer with 2. The CNN network is used for the MNIST classification task, which contains two convolutional layers and two fully connected layers.

**Configurations: **All of the models use Adam as the optimizer with a learning rate of 1e–4 and a batch size of 64. The client and server run on different TITAN Xp graphics cards for federated learning, and all models upload parameters once every two epochs of training locally on the client. The server aggregates the parameters via the FedAvg algorithm, where the total number of communications during the training of the VAE model is 78, and the total number of communications during the training of the VGG16 model is 50, the number of global communication rounds for the CNN model is 100.

### Experimental results

#### Exploratory experiments on FedDIS

This experiment was performed on the MRI dataset. Three types of distributions, including ordinary normal, truncated normal, and uniform distribution types, were used to estimate the distribution information in the hidden space, and data augmentation was performed on the client. After the data generation was completed, the final diagnostic classification experiment was performed.

**Fig. 6:**
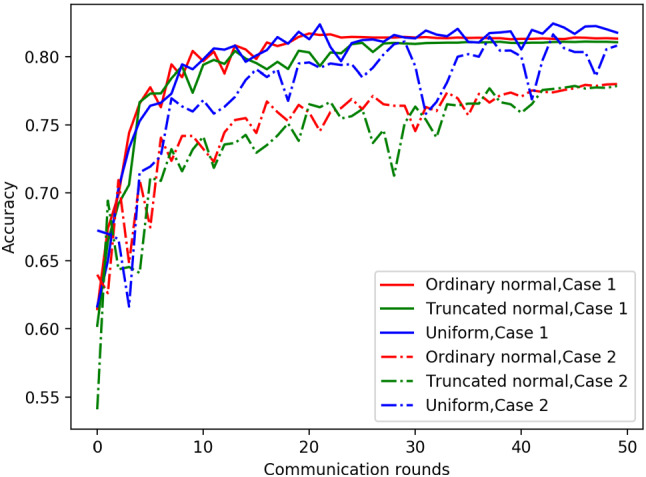
Test accuracy over communication rounds using different distribution types

To explore the effect of different hidden space distribution types on the experimental results, Fig. 6 shows the test accuracy over communication rounds using different distribution types in different cases when the $\sigma$-point parameter $\alpha$ is fixed to 1. It can be clearly found that the test performance is better for data with a non-IID degree. different EMD cases when the distribution type is uniform, which indicates that the uniform distribution type is more suitable for the current dataset.

**Fig. 7:**
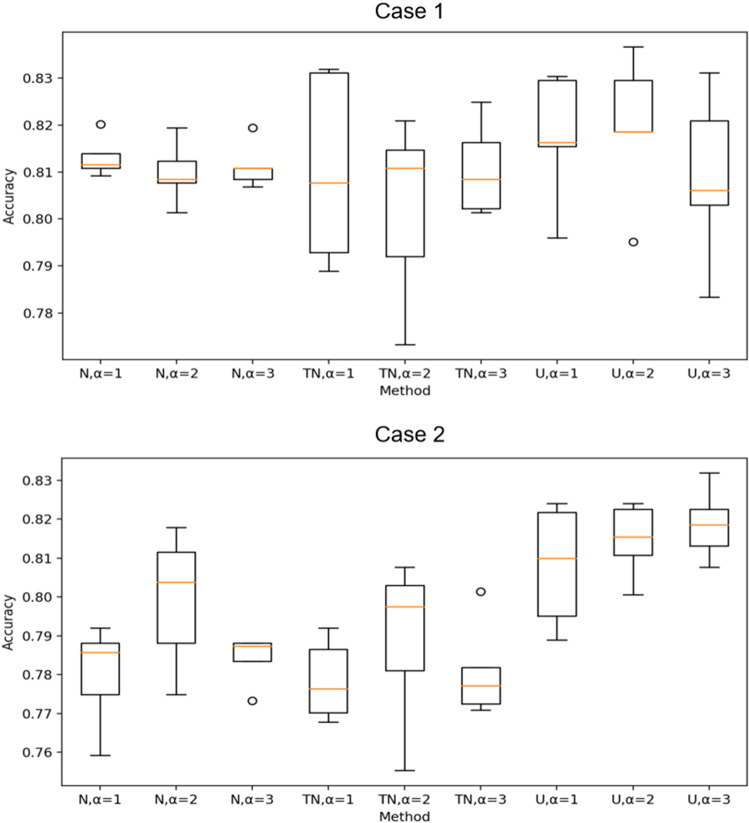
Boxplots of test accuracy in different cases. N, TN, and U represent ordinary normal distribution, truncated normal distribution and uniform distribution types, respectively

To better show the effect of different hidden space distribution types and $\sigma$-point parameters on the final test performance, Fig. 7 shows the boxplots of the test accuracy across 5 runs using different distribution types in different cases with different $\sigma$-point parameters. As we can see, different hidden space distribution types and different $\sigma$-point parameters cause differences in the final results, and the performance is better using the uniform distribution type and $\alpha =2$.

#### Comparison experiments

This experiment consisted of two comparisons, the first performed on the MRI dataset with client-side local data imbalance. FedDIS is compared with the FedAvg algorithm [12], the data sharing (DS) [16] and data redundancy (DR) [17] approaches, and the synthetic minority oversampling technique (SMOTE) was used to generate minority class data locally based on the distribution of local data as a comparison. SMOTE has been applied to the medical data imbalance problem [27], and we use the SMOTE technique on the client with a non-IID data problem, followed by federated learning and testing its performance for the three clients. We call this method Fed_SMOTE. For a fair comparison, experiments were conducted on the same dataset and in the exact same environment, including the following method setup.

- Create a global shared dataset with a class uniform distribution, $\beta$ set to 5% and 10%, respectively, where $\beta =(||G||/||D||)\times 100\%$, *G* represents the size of the global shared dataset and *D* represents the size of the total dataset, a random $\alpha$ portion of *G* are distributed to each client, and $\alpha$ is set to 100%, i.e., the global shared dataset is fully allocated to each client.
- Data redundancy is introduced in the system, where the data redundancy is set to *r* = 2, representing that each copy of data is stored on 2 different clients.

**Fig. 8:**
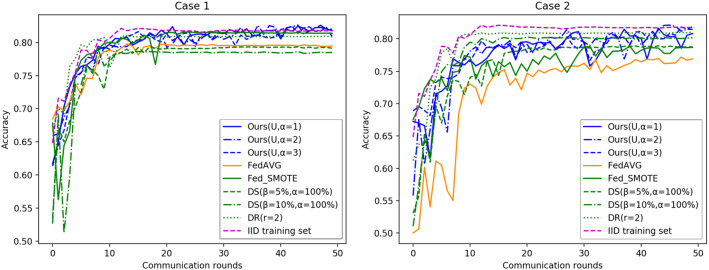
Test accuracy of different methods over communication rounds

**Fig. 9:**
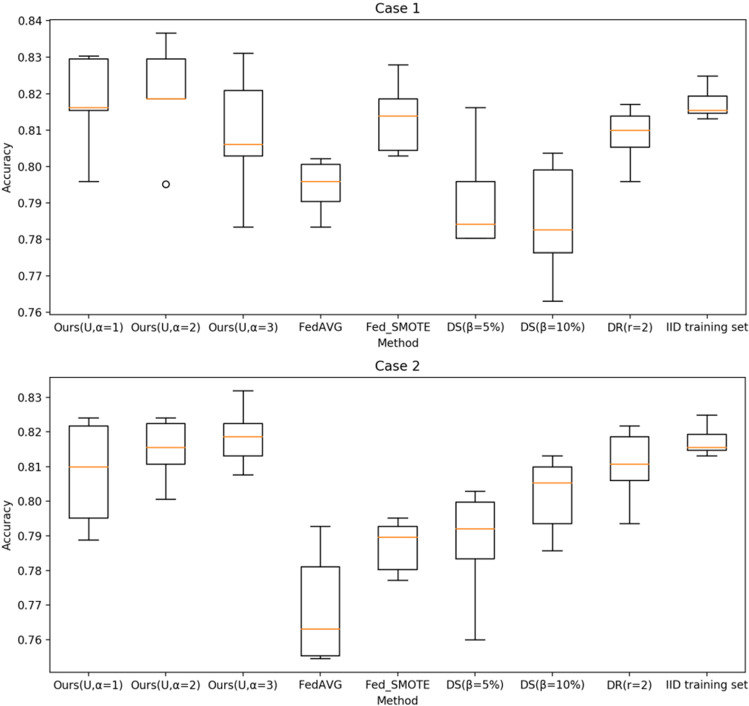
Boxplots of test accuracy in different cases for different methods

The results directly on the IID training set are used as a target reference to better reflect the performance of the methods. Figure 8 shows the test accuracy of the proposed method and method of comparison as the number of communication rounds increases. To take a closer look at the variations, the boxplots show the test accuracy across 5 runs in different cases in Fig. 9. The experimental results demonstrate the effectiveness of FedDIS. We also observe a rapid decrease in the effectiveness of the Fed_SMOTE method when the data imbalance increases.

**Fig. 10:**
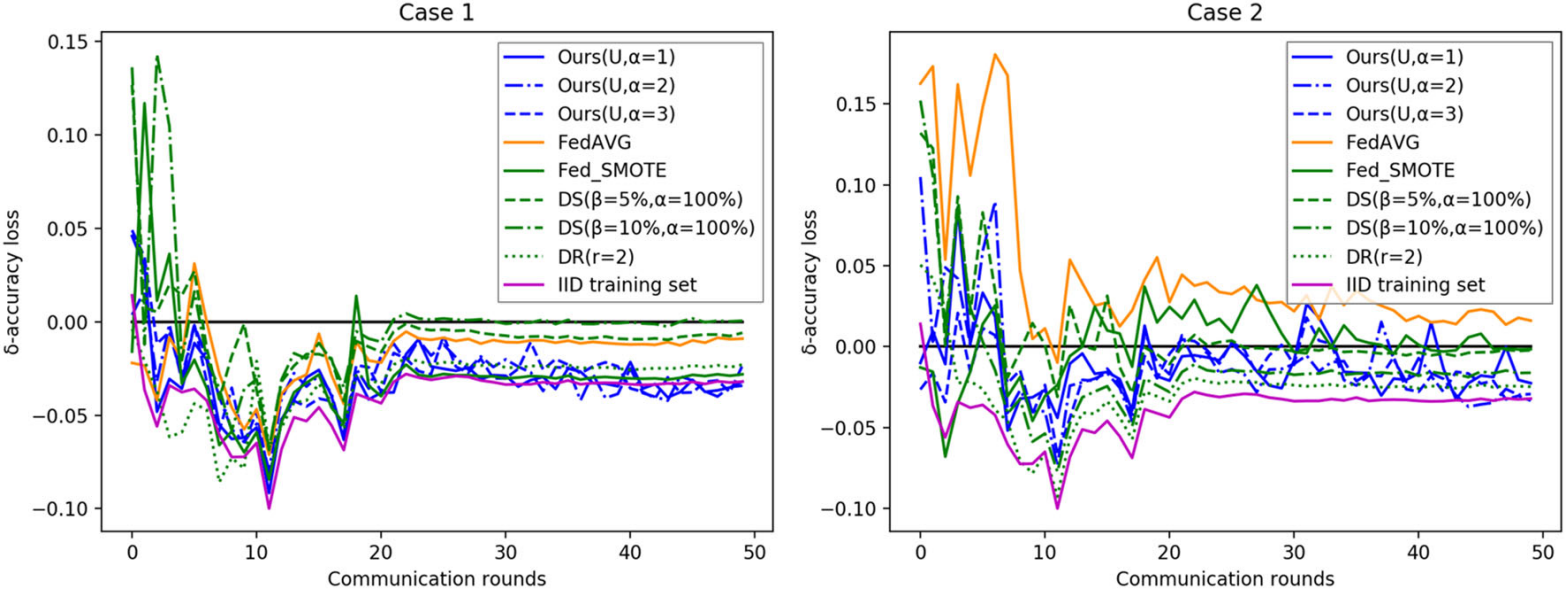
$\delta$-Accuracy loss of different methods over communication rounds

To more visually measure the difference in performance between federated and centralized learning, the $\delta$-accuracy loss [28] is improved by subtracting the test accuracy of the centralized learning from the test accuracy of the federated learning method to better discover which of the two performs better. The $\delta$-accuracy loss curve for each method with an increasing number of communication rounds is shown in Fig. 10. Since the proposed method generates new training data in the process of balancing the dataset, which gives the method more training data than centralized learning, the trained model has more generalization ability and, therefore, $\delta$ accuracy loss will appear to be less than zero.

Tables 5 and 6 show the final test performance of the different methods in different non-IID cases. The comparison results show that the final classification accuracy of FedDIS under the best setting is higher than that of the comparison method with performance comparable to training directly on the IID dataset. And FedDIS does not share or exchange the original data, so there is no problem of privacy leakage of the original data. Therefore, FedDIS is more secure and more in line with the requirements of federated learning.

**Table 5:** Test accuracy using different methods in case 1

Method	Accuracy (%)
FedAvg	79.43
Fed_SMOTE	81.37
DS ($\beta$ = 5%, $\alpha$ = 100%)	79.21
DS ($\beta$ = 10%, $\alpha$ = 100%)	78.49
DR (*r* = 2)	80.89
IID training set	81.75
FedDIS under the best setting	**82**.**17**

The bold number indicates the optimal performance

**Table 6:** Test accuracy using different methods in case 2

Method	Accuracy (%)
FedAvg	76.95
Fed_SMOTE	78.68
DS ($\beta$ = 5%, $\alpha$ = 100%)	78.75
DS ($\beta$ = 10%, $\alpha$ = 100%)	80.10
DR (*r* = 2)	80.97
IID training set	**81**.**75**
FedDIS under the best setting	81.74

The bold number indicates the optimal performance

The second comparison experiment is executed on the MNIST dataset with non-IID among clients, the baseline for comparison is FedAvg [12]. FedProx is a typical algorithm-level approach to non-IID problems by making corrections to the gradient [10], and is a representative algorithm for correcting the local model with the global model. FEDFUSION [13] and FEDGEN [14] are typical data-level methods for data-based and data-free, respectively. methods, which are the two mainstream methods for solving non-IID problems at the data level. We run three trials and report the Top-1 test accuracy. Table 7 presents the comparison results, from the results we can see that FedDIS has a better performance compared to other methods and is more superior in the more extreme non-IID cases.

**Table 7:** Top-1 accuracy of FedDIS and the other baselines on test dataset

$\beta$	0.05	0.1	1
FedAvg	87.70 ± 2.07	90.16 ± 0.59	93.84 ± 0.25
FedProx	87.49 ± 2.05	90.10 ± 0.39	93.83 ± 0.29
FEDFUSION	90.02 ± 0.96	91.11 ± 0.43	93.37 ± 0.40
FEDGEN	91.30 ± 0.74	93.03 ± 0.32	95.52 ± 0.07
FedDIS	**95.16 ± 0.05**	**95.69 ± 0.53**	**97.13 ± 0.03**

The bold numbers indicate the optimal performance

**Fig. 11:**
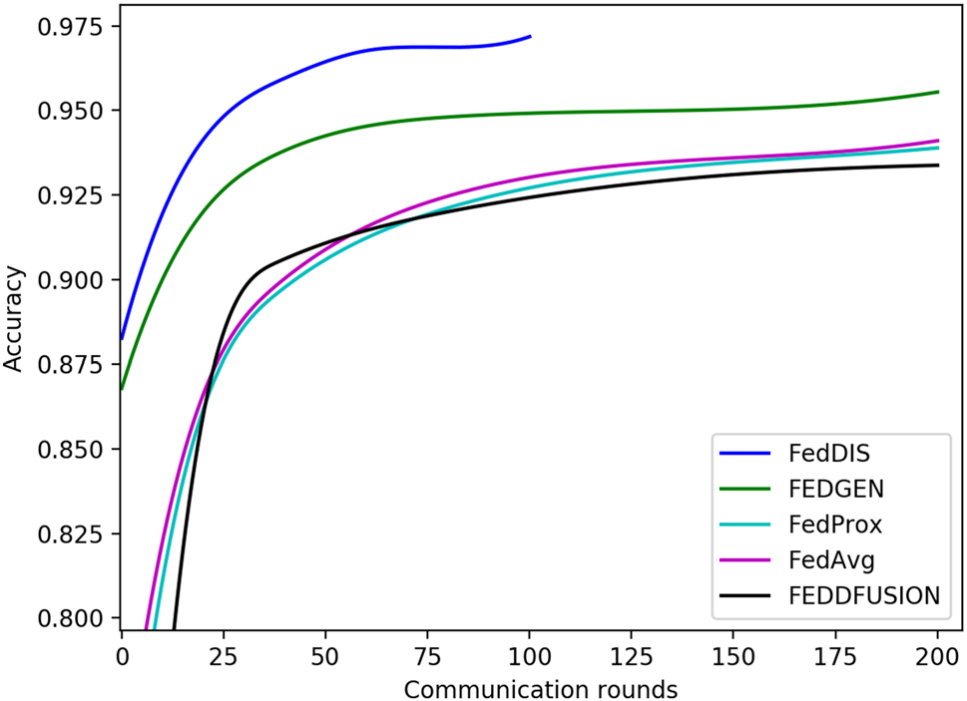
Test accuracy curves for different methods, $\beta$=1

As shown in Fig. 11, FedDIS has the fastest convergence speed and achieves a better performance than the other methods. FEDGEN uses the generator to guide the user model during training the generator. Due to the poor quality of the generator at the beginning of training, it does not regulate the local model well, while FedDIS trains a generator independently first and reduce non-IIDness across clients before training the task model, so it has a faster convergence speed when training the task model, and because we improve the hidden space of VAE to generate higher quality data locally, FedDIS eventually achieves higher accuracy.

## Conclusion and future work

In this paper, we introduced FedDIS to image classification, including the detailed design and implementation process, which can share the distribution information of the hidden space data under the condition of privacy protection and thus indirectly share the distribution information of the medical images. Using this distribution information, we can perform data generation processing for clients with non-IID data to reduce non-IIDness across clients, thus improving the performance of image classification. We conducted experiments on the Alzheimer’s disease MRI image dataset and MNIST dataset, two types of experiments were done, first on the algorithm itself, including exploring the effect of different hidden space distribution types and $\sigma$-point parameters on the performance of the method. Then comparative experiments were done, under the condition of local data imbalance on the client side and under the condition of inconsistent data distribution among the clients, which effectively verified the superiority of the method. In addition, the hidden space distribution information sharing strategy can be used as a new method for sharing data knowledge in privacy-preserving situations, which has positive implications for improving the performance of traditional federated learning. For further research, we will explore ways to improve the federated learning performance in the case of heterogeneous image data from different institutions by considering the mapping of heterogeneous data to homogeneous data as an image translation problem, which can be handled using, for example, vision transformer and adversarial network models.

## Data Availability

The Alzheimer disease dataset is publicly accesible at https://www.kaggle.com/legendahmed/alzheimermridataset
